# Controllable Sequence Editing for Biological and Clinical Trajectories

**Published:** 2025-06-03

**Authors:** Michelle M. Li, Kevin Li, Yasha Ektefaie, Ying Jin, Yepeng Huang, Shvat Messica, Tianxi Cai, Marinka Zitnik

**Affiliations:** 1 Harvard University; 2 MIT

## Abstract

Conditional generation models for longitudinal sequences can generate new or modified trajectories given a conditioning input. While effective at generating entire sequences, these models typically lack control over the timing and scope of the edits. Most existing approaches either operate on univariate sequences or assume that the condition affects all variables and time steps. However, many scientific and clinical applications require more precise interventions, where a condition takes effect only after a specific time and influences only a subset of variables. We introduce Clef, a controllable sequence editing model for conditional generation of immediate and delayed effects in multivariate longitudinal sequences. Clef learns temporal concepts that encode how and when a condition alters future sequence evolution. These concepts allow Clef to apply targeted edits to the affected time steps and variables while preserving the rest of the sequence. We evaluate Clef on 7 datasets spanning cellular reprogramming and patient health trajectories, comparing against 9 state-of-the-art baselines. Clef improves immediate sequence editing accuracy by up to 36.01% (MAE). Unlike prior models, Clef enables one-step conditional generation at arbitrary future times, outperforming them in delayed sequence editing by up to 65.71% (MAE). We test Clef under counterfactual inference assumptions and show up to 63.19% (MAE) improvement on zero-shot conditional generation of counterfactual trajectories. In a case study of patients with type 1 diabetes mellitus, Clef identifies clinical interventions that generate realistic counterfactual trajectories shifted toward healthier outcomes.

## 1 Introduction

As the US Food and Drug Administration (FDA) phases out animal testing requirements for therapeutic discovery, AI tools will become widely adopted to simulate the effects of candidate drugs [19]. In particular, building virtual cells and patients to model the behavior of molecules, cells, and tissues could facilitate large-scale *in silico* experimentation, such as approximating the efficacy of a drug [8, 45]. In this work, we focus on the conditional generation of longitudinal sequences. **We posit that conditional sequence generation models can be used to infer potential outcomes under specific conditions:** *Generate a cell’s state after treating it with a candidate drug every hour or every 24 hours. Generate a patient’s state after performing surgery today or next year*. To successfully build virtual cells and patients, we must be able to reason about both the choice of the intervention (e.g., drug, surgery) and its timing (e.g., when and how frequent). Thus, conditional generation on longitudinal sequences requires precise and context-specific edits. For example, prescribing a medication to a patient should result in changes to the patient’s trajectory only after the intervention time (i.e., the medical history prior to intervention should be unaffected) and on only the relevant variables that are specific to the context of the intervention (i.e., the measurements unaffected by the intervention should be preserved).

Generative language and vision models enable precise editing guided by a description, such as textual prompts or condition tokens [101, 20, 79, 23, 72, 25, 103]. These models are designed to gain more *global* context-preserving and *local* precise control over the generation of text [10, 72, 25, 103], images [101, 20, 79], and even molecular structures [23, 12, 102]. Their outputs preserve the input’s global integrity yet contain precise local edits to satisfy the desired condition. Analogous to these models’ consideration of spatial context to edit images [101, 20] and protein pockets [12, 102] via in-painting, **our work leverages temporal context to edit sequences based on a given condition**.

Controllable text generation (CTG) approaches, designed specifically to edit natural language sequences, have been extensively studied [99]. **They excel in *immediate sequence editing***: predicting the next token or readout in the sequence under a given condition [72, 25, 103, 10, 99, 6]. For example, if asked to predict the next word in the sentence “Once upon a time, there lived a boy” under the condition that the genre is horror, a CTG model may respond with “alone” to convey vulnerability and loneliness. However, **CTG models are unable to perform *delayed sequence editing*:** modifying a trajectory in the far-future. The distinction is important: the focus is on when the edit occurs, not necessarily when its effects manifest. Whereas immediate sequence editing applies a condition now (e.g., administering insulin *today*), delayed sequence editing schedules a condition for the *future* (e.g., starting a chemotherapy regimen *in six* weeks). Existing CTG models cannot effectively utilize the given context to skip ahead to the future; instead, they would need to be run repeatedly to fill in the temporal gap without any guarantee of satisfying the desired condition. As a result, **CTG models are insufficient for other sequence types (i.e., not natural language) for which both immediate and delayed sequence editing are necessary**, such as cell development and patient health trajectories.

There exist two controllable time series generation (CTsG) approaches [33, 4], which utilize diffusion modeling to generate time series under a given condition. However, they are limited to univariate sequences and assume that the entire input sequence is affected [33, 4]. These methods are thus insufficient in settings where edits are only allowed after time t (i.e., cannot change historical data) and affect only certain sequences (i.e., preserve unaffected co-occurring sequences). In other words, **CTsG methods are unable to make precise local edits while preserving global integrity**. Orthogonal to CTsG is the estimation of counterfactual outcomes over time (ECT) [53, 7, 29, 88]. Although not generative, ECT autoregressively predicts the potential outcomes (i.e., next readout in the sequence) as a result of different future treatments (i.e., fixed set of conditions) under counterfactual inference assumptions. **While ECT preserves historical and unaffected co-occurring sequences, counterfactual inference assumptions may not always hold in real-world applications.**

### Present work.

We develop Clef^1^, a novel ControLlable sequence Editing Framework for instance-wise conditional generation. Clef learns temporal concepts that represent the trajectories of the sequences to enable accurate generation guided by a given condition (Definition 3.2). We show that the learned temporal concepts help preserve temporal constraints in the generated outputs. By design, Clef is flexible with any type of sequential data encoder. We demonstrate through experiments on 4 new benchmark datasets in cellular reprogramming and patient immune dynamics that Clef outperforms state-of-the-art models by up to 36.01% and 65.71% (MAE) on immediate and delayed sequence editing (Definition 3.1). We also show that any pretrained sequence encoder can gain controllable sequence editing capabilities when finetuned with Clef. Additionally, we extend Clef for multi-step ahead counterfactual prediction under counterfactual inference assumptions (Assumption 3.4, Equation 5), and demonstrate (on 3 benchmark datasets) performance gains against 5 state-of-the-art baselines in settings with high time-varying confounding. Moreover, Clef enables conditional generation models to outperform baselines in zero-shot generation of counterfactual cellular trajectories by up to 14.45% and 63.19% (MAE) on immediate and delayed sequence editing. Further, precise edits via user interaction can be performed directly on Clef’s learned concepts. We show through real-world case studies that Clef, given precise edits on specific temporal concepts, can generate realistic “healthy” trajectories for patients originally with type 1 diabetes mellitus.

### Our contributions are fourfold.

(1) We develop Clef: a flexible controllable sequence editing model for conditional generation of longitudinal sequences. (2) Clef can be integrated into the (balanced) representation learning architectures of counterfactual prediction models to estimate counterfactual outcomes over time. (3) Beyond achieving state-of-the-art performance in conditional sequence generation and counterfactual outcomes prediction, Clef excels in zero-shot conditional generation of counterfactual sequences. (4) We release four new benchmark datasets on cell reprogramming and patient immune dynamics for immediate and delayed sequence editing, and evaluate on three established benchmark datasets regarding patient tumor growth for counterfactual prediction.

## 2 Related work

### Sequence editing.

The sequence editing task has been defined in language and time series modeling via different terms, but share a core idea: Given a sequence and a condition (e.g., sentiment, attribute), generate a sequence with the desired properties. Conditional sequence generation is an autoregressive process in language [10] but a diffusion process in time series [33, 4]. Prompting is often used to guide the generation of a sequence, both textual and temporal, with a desired condition [99, 6, 33, 4]. However, existing approaches are unable to generate multivariate sequences, preserve relevant historical data, and ensure time-sensitive edits. They assume that sequences are univariate and conditions affect the entire sequence [33, 4]. Structural causal models can be incorporated to enable counterfactual text generation while preserving certain attributes [10, 78]. Estimating counterfactual outcomes over time is often formulated under the potential outcomes framework [55, 82].

### Estimating counterfactual outcomes over time.

Predicting time-varying counterfactual outcomes in time series entails estimating counterfactual outcomes over possible sequences of interventions, including the timing and ordering of sequential treatments [53, 7, 29, 88]. There are decades of research on temporal counterfactual outcomes estimation [81, 46, 7, 44, 53]. Recently, machine learning approaches for predicting time-varying counterfactual outcomes learn representations that are predictive of outcomes while mitigating treatment bias via balancing techniques [53, 7, 29, 88]. On image data, conditional generation models (i.e, guided diffusion, conditional variational autoencoder) have been shown to predict counterfactual outcomes without an explicit density estimation [93]. However, there may be a trade-off between prediction accuracy and balanced representations [29].

### Concept-based learning.

Concepts can be thought of as abstract atomic ideas or concrete tokens of text or images [86, 43]. Concept-based learning has been used to explain (e.g., predict the concepts observed in the sample) or transform black-box models into more explainable models (e.g., allow users to intervene on learned concepts) [39, 84, 31, 43, 42, 85]. While concepts have been used in sequence generation [86], they have not yet been used for conditional generation of longitudinal sequences. The adoption of concept-based learning for counterfactual prediction is limited to improving the interpretability of image classification [16, 13, 15].

Refer to Appendix A for further discussion of related work.

## 3 Clef

Clef manipulates sequences based on user-specified conditions and temporal coordinates. Given a longitudinal sequence, forecast time step, and condition token, Clef modifies only the relevant portions of the sequence while preserving unaffected elements, ensuring global integrity. Architecturally, Clef has four key components: **(i) sequence encoder** F that extracts temporal features from historical sequence data, **(ii) condition adapter** H that maps condition tokens to latent representations, **(iii) concept encoder** E that learns temporal concepts, representing trajectory patterns over time, and **(iv) concept decoder** G that applies these concepts to generate sequences.

### 3.1 Problem definition

Consider an observational dataset $𝒟={\left\{x_{t}^{\left(i\right)},s_{t}^{\left(i\right)}\right\}}_{i=1}^{N}$ for N independent entities (e.g., cells, patients) at time step t. For each entity i at time t, we observe continuous time-varying covariates $x_{t}^{\left(i\right)}\in R^{d_{x}}$ (e.g., gene expression, laboratory test measurements) and categorical conditions $s_{t}^{\left(i\right)}$ (e.g., transcription factor activation, clinical intervention). The outcome of the condition is measured by the covariates (e.g., a transcription factor activation affects a cell’s gene expression, a medication affects a patient’s laboratory test profile). For notation, we omit entity index (i) unless needed.

**Figure 2:**
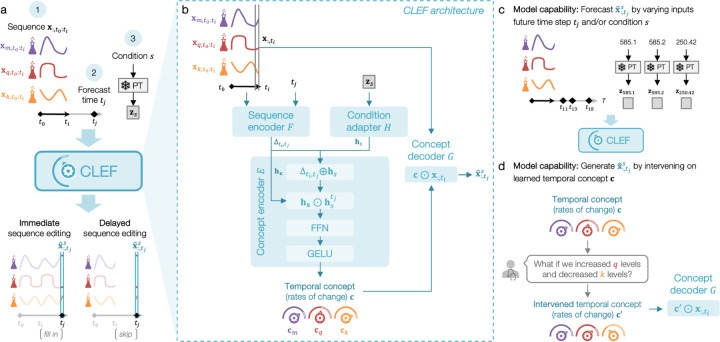
Overview of Clef’s architecture and capabilities. **(a)** Given a sequence, forecast time, and condition embedding from a frozen pretrained (PT) embedding model, Clef generates a sequence via immediate or delayed sequence editing. **(b)** Clef is composed of a sequence encoder, condition adapter, concept encoder, and concept decoder. Clef has two key capabilities: **(c)** forecast sequences at any future time and under any condition (e.g., medical codes), and **(d)** generate sequences by intervening on CleF’s learned temporal concepts.

**Definition 3.1** (Sequence editing). Sequence editing is the local sample-level modification of sequence x to autoregressively generate ${\overset{ˆ}{x}}_{:,t_{j}}$ under condition s given at time $t_{j}-\epsilon$. Time gap ϵ indicates that ${\overset{ˆ}{x}}_{:,t}$ is measured a negligible amount of time after s is applied^2^; for notation, we omit ϵ unless needed. There are two types of controllable sequence editing (Figure 2a):

- **Immediate sequence editing:** Given $x_{:,t_{0}:t_{i}}$ and s to occur at $t_{i+1}$, forecast ${\overset{ˆ}{x}}_{:,t_{i+1}}$
- **Delayed sequence editing:** Given $x_{:,t_{0}:t_{i}}$ and s to occur at $t_{j}\geq t_{i+1}$, forecast ${\overset{ˆ}{x}}_{:,t_{j}}$

Examples of immediate sequence editing include generating trajectories after perturbing cells *now* or performing surgery on patients *today* (Section 5.1). In contrast, delayed sequence editing generates trajectories after perturbing cells *in ten days* or performing surgery on patients *next year* (Section 5.2).

**Definition 3 .2** (Temporal concept). Temporal concept c is defined by $c=x_{:,t_{k}}/x_{:,t_{j}}$ for sequence x where time steps $t_{k}>t_{j}$. It can be interpreted as the trajectory (or rate of change of each variable in the sequence) between any pair of time steps.

**Definition 3.3** (Controllable sequence editing). Concept encoder E and decoder G can leverage temporal concepts c to perform controllable sequence editing if the following are satisfied.

- Condition s on $x_{:,t_{0}:t_{i}}$ at time step $t_{j}$ learns c that accurately forecasts ${\overset{ˆ}{x}}_{:,t_{j}}^{s}$ such that ${\overset{ˆ}{x}}_{:,t_{j}}^{s}≃x_{:,t_{j}}^{s}$.
- For an alternative condition a≠s on $x_{:,t_{0}:t_{i}}$ at $t_{j}$, the method learns a distinct $c^{\prime }\neq c$ that forecasts ${\overset{ˆ}{x}}_{:,t_{j}}^{a}$ such that ${\overset{ˆ}{x}}_{:,t_{j}}^{a}\neq {\overset{ˆ}{x}}_{:,t_{j}}^{s}$ and, if known, ${\overset{ˆ}{x}}_{:,t_{j}}^{a}≃x_{:,t_{j}}^{a}$.

#### Problem Statement 3.1 (Clef).

Given a sequence encoder F, condition adapter H, concept encoder E, and concept decoder G trained on a longitudinal dataset 𝒟, Clef learns temporal concept $c=E\left(F\left(x_{:,t_{0}:t_{i}},t_{j}\right),H(s)\right)$ to forecast ${\overset{ˆ}{x}}_{:,t_{j}}^{s}=G\left(x_{:,t_{i}},c\right)$ for any $x_{:,t_{0}:t_{i}}\in 𝒟,t_{j}>t_{i}$, and s.


(1) $${\overset{ˆ}{x}}_{:,t_{j}}^{s}=G\left(x_{:,t_{i}},E\left(F\left(x_{:,t_{0}:t_{i}},t_{j}\right),H(s)\right)\right)$$

### 3.2 Clef architecture

The input to Clef is a continuous multivariate sequence $x_{:,t_{0}:t_{i}}\in R^{V}$ with V measured variables, a condition s, and time $t_{j}>t_{i}$ for which to forecast ${\overset{ˆ}{x}}_{:,t_{j}}^{s}$. Clef consists of four major components: a sequence encoder F, a condition adapter H, a concept encoder E, and a concept decoder G.

#### Sequence encoder F.

The sequence encoder F extracts features $x_{:,t_{0}:t_{i}}$ such that $h_{x}=F\left(x_{:,t_{0}:t_{i}}\right)$. Any encoder, including a pretrained multivariate foundation model, can be used. The time encoder in F generates a time positional embedding $h_{t}$ for any time t via element-wise summation of the year (sinusoidal), month, date, and hour embeddings. It is also used to compute the time delta embedding $\Delta_{t_{i},t_{j}}=h_{t_{j}}-h_{t_{i}}$ for the concept encoder E.

#### Condition adapter H.

The condition token, or embedding $z_{s}$ corresponding to the input condition s, is retrieved from a frozen pretrained embedding model (denoted as pt in Figure 2a). The condition adapter H projects $z_{s}$ into a hidden representation $h_{s}=H(s)$.

#### Concept encoder E.

Given the hidden representations generated by sequence encoder F and condition adapter H, concept encoder E learns temporal concept $c=E\left(h_{x},\Delta_{t_{i},t_{j}},h_{s}\right)$. First, the time delta embedding $\Delta_{t_{i},t_{j}}$ is combined via summation with the condition embedding $h_{s}$ to generate a time- and condition-specific embedding $h_{s}^{t_{j}}=\Delta_{t_{i},t_{j}}⊕h_{s}$. Temporal concept c is learned via an element-wise multiplication of $h_{x}$ and $h_{s}^{t_{j}}$, an optional linear projection using a feedforward neural network (FNN), and a GELU activation to approximate the trajectory between $t_{i}$ and $t_{j}$

(2) $$c=\text{GELU}\left(\text{FFN}\left(h_{x}⊙h_{s}^{t_{j}}\right)\right)$$

#### Concept decoder G.

The concept decoder G forecasts ${\overset{ˆ}{x}}_{:,t}^{s}$ by performing element-wise multiplication of the latest time $t_{i}$ of the input sequence $x_{:,t_{0}:t_{i}}$ (denoted as $x_{:,t_{i}}$) and the learned concept c

(3) $${\overset{ˆ}{x}}_{:,t_{j}}^{s}=c⊙x_{:,t_{i}}$$

#### Objective function ℒ.

Sequence editing loss function ℒ quantifies the reconstruction error of the predicted ${\overset{ˆ}{x}}_{:,t_{j}}^{s}$ and the ground truth $x_{:,t_{j}}^{s}$. Here, we use Huber loss, where $a=x_{:,t_{j}}^{s}-{\overset{ˆ}{x}}_{:,t_{j}}^{s}$ and δ=1,

(4) $$ℒ\left(x_{:,t_{j}}^{s},{\overset{ˆ}{x}}_{:,t_{j}}^{s}\right)=\left\{\begin{array}{ll} 0.5a^{2}, & \text{if}|a|\leq \delta \\ \delta (|a|-0.5\delta ), & \text{otherwise} \end{array}\right.$$

### 3.3 Clef’s connection to counterfactual prediction

Let $x_{:,t_{j}}$ refer to the outcomes observed at $t_{j}$ after treatment s is given. Our problem can be viewed as counterfactual prediction when there is no treatment assigned between $t_{i}$ and $t_{j}$ except s.

Formally, under the potential outcomes framework [55, 82] and its extension to time-varying treatments and outcomes [80], the potential counterfactual outcomes over time are identifiable from the observational data 𝒟 under three standard assumptions: consistency, positivity, and sequential ignorability (Appendix B). Thus, Clef predicts counterfactuals under the additional Assumption 3.4:

**Assumption 3.4** (Conditional mean function estimation). For time steps $t_{j}>t_{i}$, temporal concepts c learned based on the next treatment $s_{t_{j}}$, historical treatments $s_{t_{0}:t_{i}}$, and historical covariates $x_{:,t_{0}:t_{i}}$ capture (balanced) representations such that the concept decoder $c\left(s_{t_{j}},s_{t_{0}:t_{i}},x_{:,t_{0}:t_{i}}\right)⊙x_{:,t_{i}}$ approximates the conditional mean function $E\left[x_{:,t_{j}+\epsilon }\left(s_{t_{j}},s_{t_{0}:t_{i}}\right)|s_{t_{0}:t_{i}},x_{:,t_{0}:t_{i}}\right]$.

In the following, we elaborate on why it can be reasonable to view CleF as an accurate counterfactual prediction model by satisfying Assumption 3.4.

#### Estimating counterfactuals.

We estimate future counterfactual outcomes over time, formulated as

(5) $$E\left(x_{:,t_{j}+\epsilon }\left(s_{t_{j}},s_{t_{0}:t_{i}}\right)|s_{t_{0}:t_{i}},x_{:,t_{0}:t_{i}}\right)$$

by learning a function $g\left(\tau ,s_{t_{j}},s_{t_{0}:t_{i}},x_{:,t_{0}:t_{i}}\right)=G\left(x_{:,t_{i}},E\left(F\left(x_{:,t_{0}:t_{i}},t_{j}\right),H\left(s_{t_{j}}\right)\right)\right)$ with projection horizon $\tau =\left(t_{j}+\epsilon \right)-t_{i}\geq 1$ for τ-step ahead prediction (Equation 1; Section 3.2). Indeed, the key to reliable counterfactual prediction is the accurate estimation of Equation 5 to adjust for bias introduced by time-varying confounders [80]. In particular, our design of g(⋅) estimates Equation 5 well (refer to Section 5.4 for empirical results) due to the effective learning of temporal concepts (Definition 3.2) and the strong representation power of the encoders (Section 3.2).

#### Balancing representations via Clef (Appendix D.2).

Since the historical covariates and next treatment are encoded independently by F and H, the learned representations are treatment-invariant (or balanced), following the discussions in existing balanced representation learning architectures (e.g., CRN [7], CT [53]). Further, by Assumption 3.4, our designed structure isolates the causal effect of the treatment from other spurious factors, enabling reliable counterfactual estimation [100].

## 4 Experimental setup

### 4.1 Datasets

**Figure 3:**
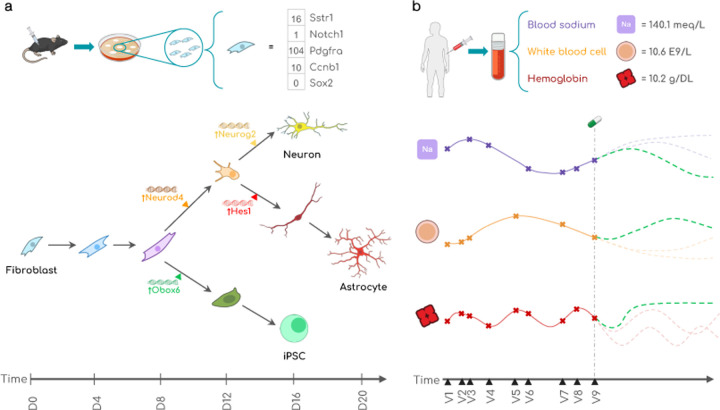
Clef is evaluated on two real-world domains involving continuous multivariate trajectories: **(a)** cellular development and **(b)** patient health. Illustrations from NIAID NIH BIOART Source (see References).

Clef is evaluated on 7 datasets in the biological and medical domains (Figure 3; Appendix C).

#### Cell development trajectories (Appendix C.1).

We introduce a new benchmarking dataset, **WOT** (Appendix Table 1). It is constructed using the Waddington-OT model, which simulates single-cell transcriptomic profiles of developmental time courses of cells [83] (Figure 3a). We also construct a new counterfactual benchmarking dataset, **WOT-CF** (Appendix Table 1). Condition tokens are defined by transcription factor embeddings generated by ESM-2 [47].

#### Patient lab test trajectories (Appendix C.2).

We construct two new real-world patient datasets of routine laboratory tests from **eICU** [75] and **MIMIC-IV** [34, 36, 22] (Figure 3b; Appendix Table 1). For benchmarking, we construct a series of data splits with different levels of train/test split similarities using SPECTRA [17] to evaluate model generalizability (Appendix Figure 10). Condition tokens are defined by embeddings of clinical codes from a clinical knowledge graph that integrates clinical vocabularies and standardized medical taxonomies used in medical health records [37].

#### Synthetic tumor growth trajectories (Appendix C.3).

We evaluate counterfactual outcomes estimation on the standard pharmacokinetic-pharmacodynamic model of tumor growth [21] for τ-step ahead prediction under the (1) **single-sliding treatment** and (2) **random trajectories** settings [7, 53]. Patient trajectories under different amounts of time-varying confounding γ are simulated [96].

#### Semi-synthetic patient trajectories (Appendix C.4).

We evaluate counterfactual outcomes estimation on a standard semi-synthetic patient dataset based on real-world medical data from intensive care units [35]. **MIMIC-III-CF** consists of patient trajectories simulated under endogenous and exogenous dependencies as well as treatment effects, enabling τ-step ahead prediction [7, 53].

### 4.2 Setup

Clef is evaluated on 3 tasks: immediate and delayed sequence editing (Definition 3.1) and counterfactual prediction (Section 3.3). We use standard metrics (MAE, RMSE, $R^{2}$) to compare ground truth $x_{:,t_{j}}^{s}$ and predicted ${\overset{ˆ}{x}}_{:,t_{j}}^{s}$. Refer to Appendix C–D for experimental setups and implementation details.

#### Baselines.

*Conditional generation* (4): We evaluate against a traditional multivariate time series model, Vector Autoregression (VAR) [51]. We benchmark against the state-of-the-art conditional sequence generation setup with different sequential data encoders: Transformer [91, 54, 33, 99] and xLSTM [5]. We further compare against a state-of-the-art time series foundation model, MOMENT [24], as the sequence encoder. *Counterfactual prediction* (5): We compare Counterfactual Recurrent Network (CRN) [7] and Causal Transformer (CT) [53] with and without balancing loss functions (i.e., gradient reversal (GR) [7], counterfactual domain confusion (CDC) [53]).

#### Ablations.

To investigate the effectiveness of the learned temporal concepts, we evaluate against an ablated model, SimpleLinear, in which temporal concepts are simply all ones; in other words, temporal concepts are not learned nor meaningful. This ablation is inspired by traditional linear models that excel when $x_{t_{j}}≃x_{t_{i}}$ [87, 1]. We also evaluate different versions of Clef with and without an FFN layer in the concept encoder E (Appendix E).

## 5 Results

We evaluate Clef’s performance on controllable sequence editing to answer the following research questions. **R1-R3:** How well does Clef perform in (R1) immediate and (R2) delayed sequence editing, and (R3) generalize to unseen/new sequences? **R4:** How does Clef perform in counterfactual outcomes estimation? **R5:** Can Clef perform zero-shot conditional generation of counterfactual sequences? **R6:** How can Clef be leveraged for real-world patient trajectory simulations? We establish that Clef outperforms state-of-the-art baselines in immediate and delayed sequence editing with strong generalizability; excels in counterfactual prediction; and demonstrates real-world applicability.

### 5.1 R1: Immediate sequence editing on observed sequences

Immediate sequence editing involves forecasting the next time step of a sequence under a given condition. This is useful in settings where interventions take effect instantaneously (Definition 3.1). Immediate sequence editing refers to scenarios where an intervention is applied at *the current time step*, such as administering a drug to a cell or performing surgery on a patient *today*.

**Figure 4:**
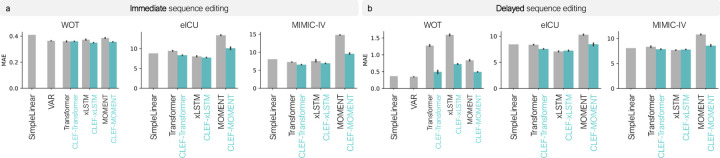
Benchmarking Clef, baselines, and ablations on **(a)** immediate and **(b)** delayed sequence editing on observed sequences. Lower MAE is better. Models are trained on 3 seeds using a standard cell- or patient-centric random split; error bars show 95% CI. Not shown for visualization purposes are VAR’s performance on eICU and MIMIC-IV: on immediate sequence editing, MAE for eICU and MIMIC-IV are 55982.74 and 886.05; on delayed sequence editing, MAE for eICU and MIMIC-IV are $3.02\times 10^{39}$ and $8.62\times 10^{23}$.

Clef models consistently outperform baseline models across all datasets (Figure 4a; Appendix Figures 8–9). Ablation SimpleLinear, which assumes no temporal changes, performs comparably in some cases, but Clef outperforms it on datasets where short-term dynamics are more complex. On WOT, all Clef models outperform or perform comparably to the time series forecasting model, VAR. This is particularly exciting given recent findings that linear models can achieve competitive or better forecasting performance than neural network models [87, 1]. These results highlight Clef’s ability to accurately edit sequences at the desired times while preserving unaffected portions of the sequence.

Regardless of the sequence encoder used with Clef, these models tend to outperform or perform comparably to non-CleF models (Figure 4a). However, Clef’s performance can be affected by the ability of the sequence encoder to capture the temporal dynamics of the input sequences. For instance, models with the MOMENT encoder generally yield the highest MAE in all three datasets (Figure 4a). Still, Clef-MOMENT models have lower MAE than their non-Clef counterparts.

### 5.2 R2: Delayed sequence editing on observed sequences

Delayed sequence editing requires forecasting a trajectory at a future time step under a given condition while maintaining global integrity. This task is challenging, as small errors can compound over longer horizons. Example scenarios in which delayed sequence editing is applicable are: Treating the cells with the candidate drug *in ten days* and performing the surgery on the patient *next year*.

Clef performs better or competitively against SimpleLinear (ablation) and VAR on eICU and MIMIC-IV (Figure 4b; Appendix Figures 8–9). Clef-transformer and Clef-xLSTM achieve lower MAE than SimpleLinear, whereas non-Clef transformer and MOMENT baselines perform comparably or worse. As in immediate sequence editing, models using MOMENT as the sequence encoder (i.e., using temporal concepts with MOMENT) yield the highest MAE. However, incorporating Clef with MOMENT reduces the MAE to levels comparable to the MAE of SimpleLinear and VAR.

On WOT, SimpleLinear and VAR outperform neural network models in delayed sequence editing (Figure 4b). This suggests that cellular trajectories exhibit small and possibly noisy changes at each time step, favoring linear models [1, 87]. Also, given the relatively small number of training trajectories compared to the high-dimensional state space, nonlinear models may overfit to noise more readily than linear models. Still, Clef significantly reduces the MAE of non-Clef models, demonstrating its effectiveness as a regularizer that mitigates short-term noise while preserving long-term trends.

### 5.3 R3: Generalization to new patient trajectories via conditional generation

We assess the Clef models’ ability to generalize to new patient sequences. To this end, we create challenging data splits where the test sets have minimal similarity to the training data (Appendix C.2) [17]. Across both the eICU and MIMIC-IV patient datasets, Clef models exhibit stronger generalization than non-Clef models (Appendix Figures 11–12 and Table 2). For immediate and delayed sequence editing on eICU, Clef-transformer and Clef-xLSTM maintain stable and strong performance even as train/test divergence increases. In contrast, their non-Clef counterparts degrade significantly. Although baseline MOMENT models show relatively stable performance across train/test splits in delayed sequence editing, they generalize poorly compared to Clef-MOMENT models. Despite similar performance between xLSTM and Clef-xLSTM in delayed sequence editing on both patient datasets (Figure 4b), Clef-xLSTM demonstrates superior generalizability (Appendix Figure 11), highlighting the effectiveness of Clef in adapting to unseen data distributions.

### 5.4 R4: Counterfactual outcomes estimation

Following the experimental setup of established benchmarks [7, 53] (Appendix D.2), we evaluate Clef on counterfactual outcomes estimation of synthetic tumor growth and semi-synthetic ICU patient (Appendix Figure 16) trajectories.

**Figure 5:**
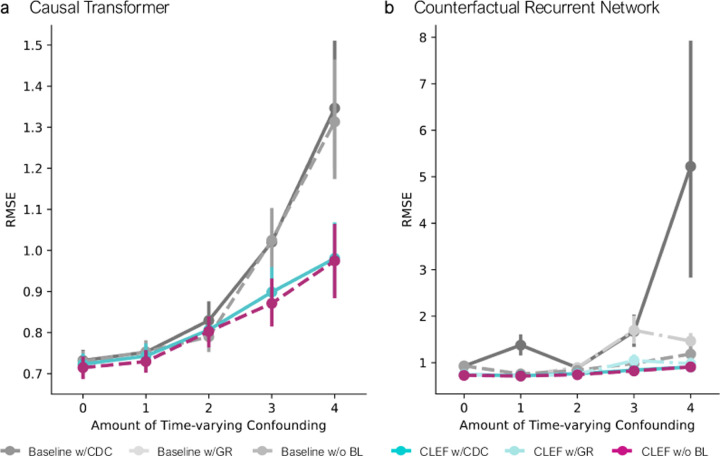
Counterfactual τ-step ahead prediction on tumor growth (single-sliding treatment) with different amounts of time-varying confounding. Models are trained on 5 seeds; error bars show 95% CI.

On the tumor growth and ICU patient trajectories, for which we have ground truth counterfactual sequences, Clef consistently performs better or competitively against non-Clef models in τ-step ahead counterfactual outcomes estimation (Figure 5; Appendix Figures 13–16). With relatively low time-varying confounding, Clef-CT with CDC loss (γ<3) and Clef-CRN with GR loss (γ<2) performs comparably to their non-Clef counterparts. When timevarying confounding is relatively high, Clef-CT with CDC loss (γ≥3) and Clef-CRN with GR loss (γ≥2) outperform their non-Clef counterparts. For all levels of confounding bias, Clef-CRN with CDC loss outperforms their non-Clef counterparts. Notably, Clef-CT and Clef-CRN without any balancing loss (i.e., neither GR nor CDC; violet-red) are the best performing CT/CRN models. While studies have shown a trade-off between prediction accuracy and balanced representations [29, 88], this finding empirically demonstrates Assumption 3.4. In other words, Clef’s strong performance without any balancing loss suggests that the temporal concepts learn balanced representations that are not predictive of the assigned treatment and approximate the conditional mean function (Equation 5; Section 3.3).

### 5.5 R5: Zero-shot conditional generation of counterfactual cellular trajectories

**Figure 6:**
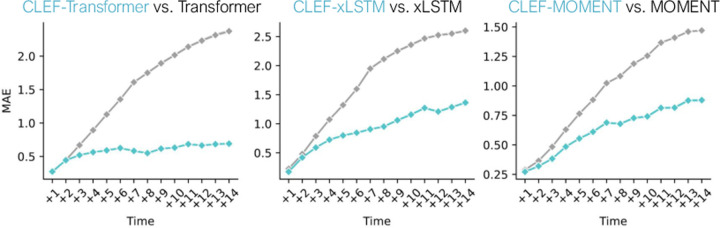
Zero-shot conditional generation of counterfactual cellular trajectories via delayed sequence editing. Shown are MAE (lower is better) of predictions per time step for counterfactual sequences of length 23 (the most common sequence length) starting at time step 10 (the earliest divergence time step of a counterfactual trajectory). Error bars show 95% CI.

We evaluate Clef on zero-shot conditional generation of counterfactual cell trajectories (Figure 6; Appendix Figure 17). Models are trained on the “original” trajectories and evaluated on the “counterfactual” trajectories in a zero-shot setting (Appendix C.1; Appendix Table 1).

Clef-based models consistently outperform non-Clef models in both immediate and delayed sequence editing (Appendix Figure 17). To more closely analyze delayed sequence editing performance, we examine the predictions for cellular trajectories of length 23, the most common sequence length in the dataset (Figure 6). Since $t_{i}=10$ is the earliest divergence time step, we input the first nine time steps $x_{:,0:9}$, the counterfactual condition, and $t_{j}\in$ [10, 23]. Comparing the generated and ground truth counterfactual sequences, we find that Clef significantly outperforms non-Clef models after time step 10, which is when the trajectories begin to diverge (Figure 6).

### 5.6 R6: Case studies using real-world patient datasets

Unlike conditional generation methods that rely on condition tokens to guide generation [54, 33, 99], CleF allows *direct edits to the generated outputs* via temporal concept intervention to produce counterfactual sequences (Appendix C.2.4). Instead of relying on predefined conditions, Clef can precisely modify the values of specific lab tests to explore their longitudinal effects. We conduct case studies on two cohorts of patients with type 1 diabetes mellitus (T1D) [77] (Appendix C.2.3).

#### Setup (Appendix C.2.4).

For each patient, we intervene on the temporal concepts corresponding to specific lab tests to simulate the “reversal” or “worsening” of symptoms, thereby generating “healthier” or “more severe” trajectories. Formally, given temporal concept c learned from $x_{:,t_{0}:t_{i}}$ and an optional condition s, we modify $c^{I}\neq c$ such that at least one element satisfies $c_{k}\neq c_{k}^{I}$. Counterfactual sequences are then simulated for T=10 (i.e., Clef-generated patients) and compared against observed sequences from matched healthy individuals, other healthy individuals, and other T1D patients. We hypothesize that clinically meaningful edits will produce “healthier” (i.e., more similar to healthy patients) or “sicker” (i.e., more similar to other T1D patients) trajectories.

#### Results.

**Figure 7:**
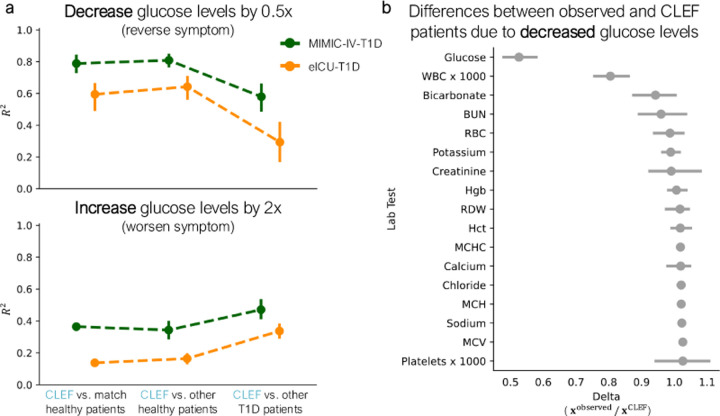
Clef-generated counterfactual patients via intervention on temporal concepts. We intervene on Clef to **(a**) halve or double a T1D patient’s glucose levels to infer a “healthier” or “sicker” counterfactual patient. Higher $R^{2}$ indicates that patient pairs are more similar. **(b)** Observed and Clef patients from the eICU-T1D cohort are compared to quantify the differences between their lab test trajectories as a result of the intervention to halve T1D patients’ glucose levels. Delta $\left(x^{\text{observed}}/x^{\text{Cler}}\right)=1$ indicates no difference between the observed and Clef patients. Error bars show 95% CI.

First, we modify Clef’s concepts to halve glucose levels, aligning them closer to normal physiological ranges. Such counterfactual trajectories exhibit higher $R^{2}$ similarity with healthy individuals compared to other T1D patients (Figure 7a), suggesting that Clef effectively generates trajectories indicative of a healthier state. Next, we simulate a worsening condition by doubling glucose levels. These trajectories generated by Clef show higher $R^{2}$ similarity with other T1D patients than with healthy individuals (Figure 7a), as would be expected based on clinical evidence.

Beyond examining the *direct effects* of the interventions on CleF’s concepts, we additionally examine the *indirect changes* in Clef-generated patients’ lab values resulting from glucose modifications. In both eICU-T1D and MIMIC-IV-T1D cohorts, lowering glucose also leads to a reduction in white blood cell (WBC) count (Figure 7b; Appendix Figure 18a). This aligns with clinical knowledge, as T1D is an autoimmune disorder where immune activity, including WBC levels, plays a critical role [77]. When we intervene on Clef to reduce WBC levels instead of glucose, glucose levels also decrease across both cohorts (Appendix Figure 18b,c), reinforcing the interdependence of these physiological markers.

Finally, we show that modifying multiple lab tests simultaneously can produce compounding effects. When we intervene on Clef to reduce both glucose and WBC levels, the resulting Clef-generated patients resemble healthy individuals even more closely than other T1D patients, suggesting that Clef can capture the joint impact of multiple simultaneous edits on a patient (Appendix Figure 18d).

## 6 Conclusion

We introduce Clef as a novel, flexible architecture that enables conditional sequence generation models to achieve state-of-the-art performance in predicting potential outcomes under specific conditions. We demonstrate that Clef excels in the conditional generation of longitudinal sequences, making precise local edits while preserving global integrity. Clef also has stronger generalizability to new sequences than state-of-the-art baselines. Moreover, under counterfactual inference assumptions, Clef accurately estimates counterfactual outcomes over time, outperforming baselines in settings with high time-varying confounding bias. Clef even outperforms state-of-the-art conditional generation models in zero-shot counterfactual generation. Further, we show that interventions directly on Clef’s temporal concepts can generate counterfactual patients such that their trajectories are shifted toward healthier outcomes. This capability has the potential to help discover clinical interventions that could alleviate a patient’s symptoms. Limitations and future directions are discussed in Appendix F. We believe that Clef’s controllable sequence editing can help realize the promise of virtual cells and patients to facilitate large-scale *in silico* experimentation of molecules, cells, and tissues [19, 8, 45].

## Figures and Tables

**Figure 1:**
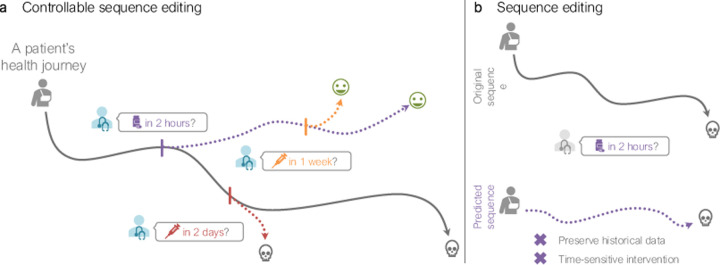
Illustrative comparison of (**a**) controllable sequence editing and (**b**) existing sequence editing. Unlike existing methods, controllable sequence editing generates sequences (dotted lines) guided by a condition while preserving historical data to model the effects of immediate (e.g., in 2 hours) or delayed (e.g., in 1 week) edits.

**Figure 8:**
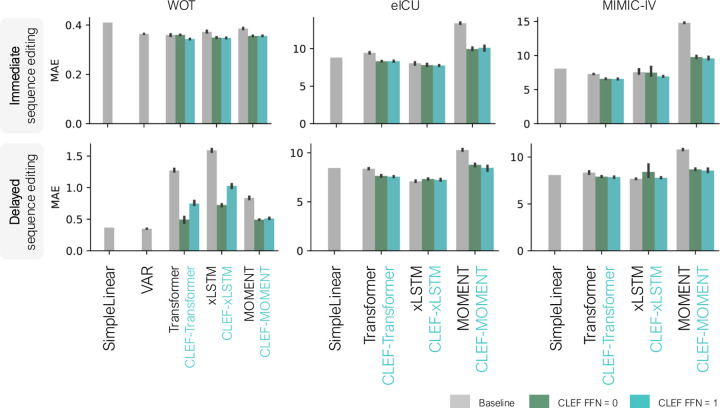
Benchmarking the performance of Clef, baselines, and ablation models on **(a)** immediate and **(b)** delayed sequence editing (Appendix C.1–C.2). Performance is measured by MAE (lower is better). Models are trained on 3 seeds using a standard cell- or patient-centric random split; error bars show 95% CI. Not shown for visualization purposes are the performances of VAR models on eICU and MIMIC-IV datasets: on immediate sequence editing, MAE for eICU and MIMIC-IV are 55982.74 and 886.05, respectively; on delayed sequence editing, MAE for eICU and MIMIC-IV are $3.02\times 10^{39}$ and $8.62\times 10^{23}$, respectively.

**Figure 9:**
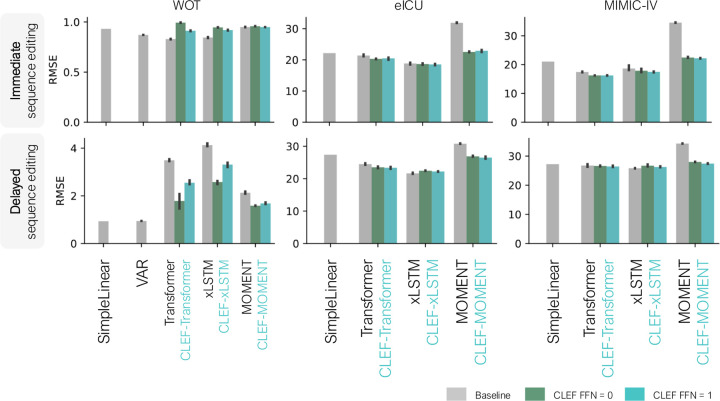
Benchmarking the performance of Clef, baselines, and ablation models on **(a)** immediate and **(b)** delayed sequence editing (Appendix C.1–C.2). Performance is measured by RMSE (lower is better). Models are trained on 3 seeds using a standard cell- or patient-centric random split; error bars show 95% CI. Not shown for visualization purposes are the performances of VAR models on eICU and MIMIC-IV datasets: on immediate sequence editing, MAE for eICU and MIMIC-IV are 135003.67 and 1793.23, respectively; on delayed sequence editing, MAE for eICU and MIMIC-IV are $5.84\times 10^{39}$ and $1.59\times 10^{24}$, respectively.

**Figure 10:**
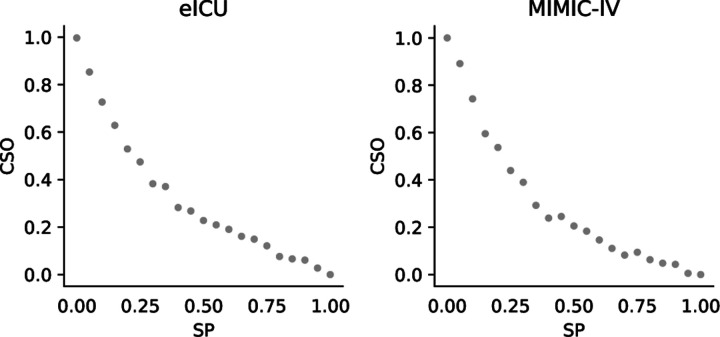
Cross-split overlap (CSO) as a function of SPECTRA parameter (SP) for eICU and MIMIC-IV datasets (Appendix C.2). CSO is defined as the number of samples in the test set that are similar to at least one sample in the train set. SP is an internal parameter used by SPECTRA to control the CSO of generated data splits. CSO decreases as SP increases. These data splits are used to evaluate conditional sequence generation models’ generalizability to unseen patient trajectories.

**Figure 11:**
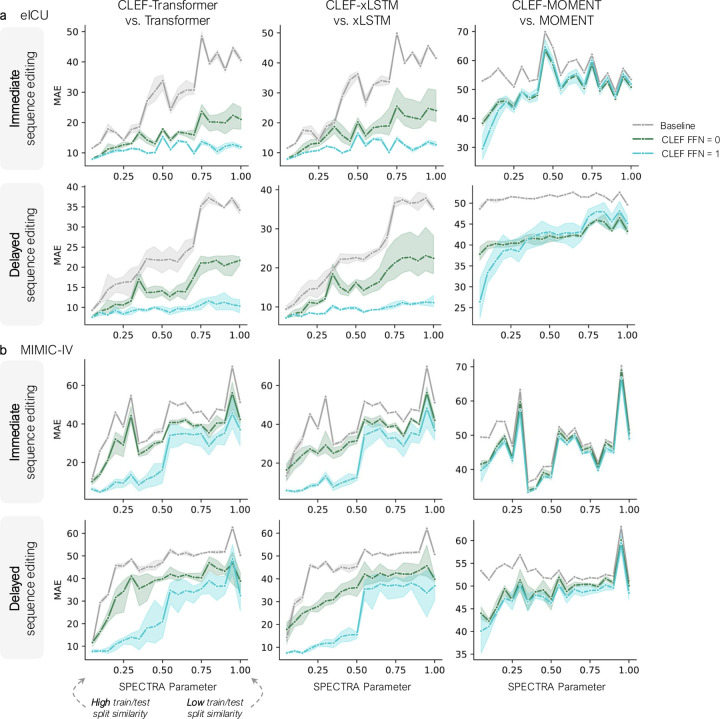
Generalizability of Clef, baselines, and ablation models on **(a**) eICU and **(b)** MIMIC-IV patient datasets (Appendix C.2) in immediate and delayed sequence editing. Performance is measured by MAE (lower is better). Models are trained on 3 seeds; error bars show 95% CI. As the SPECTRA parameter increases, the train/test split similarity decreases (Appendix Figure 10). The area under the spectral performance curve (AUSPC) evaluation is in Appendix Table 2.

**Figure 12:**
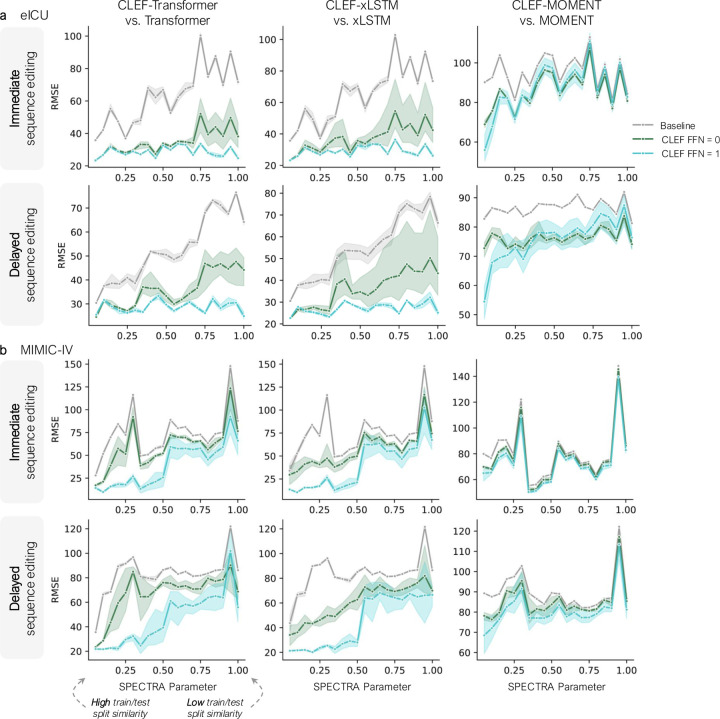
Generalizability of Clef, baselines, and ablation models on **(a)** eICU and **(b)** MIMIC-IV patient datasets (Appendix C.2) in immediate and delayed sequence editing. Performance is measured by RMSE (lower is better). Models are trained on 3 seeds; error bars show 95% CI. As the SPECTRA parameter increases, the train/test split similarity decreases (Appendix Figure 10). The area under the spectral performance curve (AUSPC) evaluation is in Appendix Table 2.

**Figure 13:**
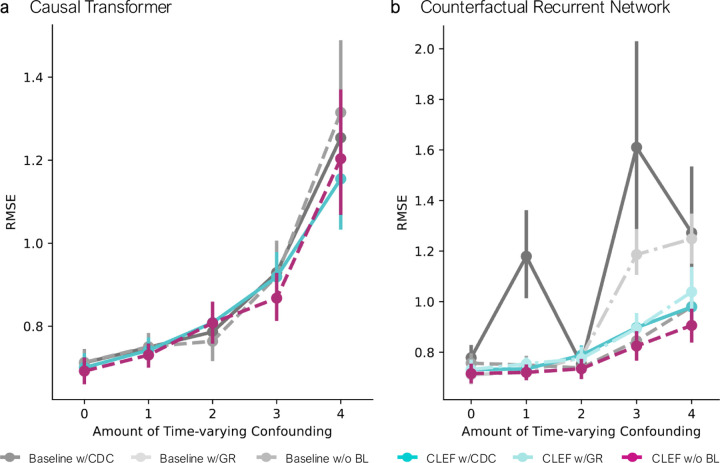
Counterfactual τ-step ahead prediction on tumor growth (random trajectories setting) with different amounts of time-varying confounding γ (Appendix C.3). GR refers to Gradient Reversal loss [7]; CDC refers to Counterfactual Domain Confusion loss [53]; BL refers to Balancing Loss (i.e., GR or CDC). Models are trained on 5 seeds; error bars show 95% CI.

**Figure 14:**
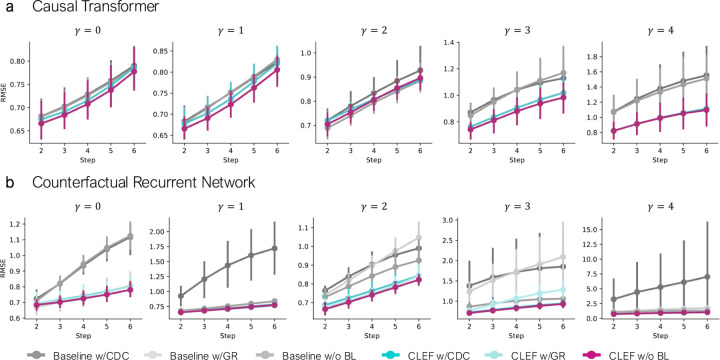
Counterfactual τ-step ahead prediction on tumor growth (single-sliding treatment) with different amounts of time-varying confounding γ (Appendix C.3). GR refers to Gradient Reversal loss [7]; CDC refers to Counterfactual Domain Confusion loss [53]; BL refers to Balancing Loss (i.e., GR or CDC). Models are trained on 5 seeds; error bars show 95% CI.

**Figure 15:**
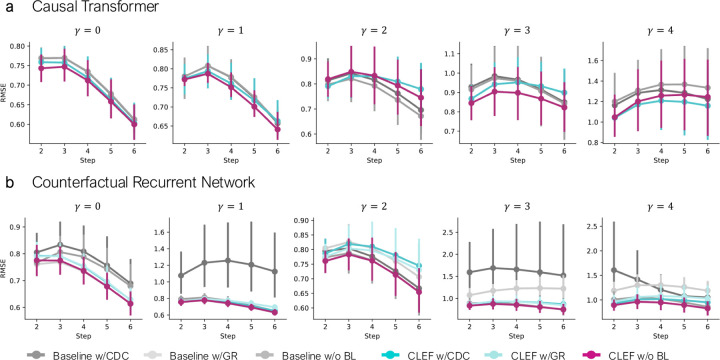
Counterfactual τ-step ahead prediction on tumor growth (random trajectories setting) with different amounts of time-varying confounding γ (Appendix C.3). GR refers to Gradient Reversal loss [7]; CDC refers to Counterfactual Domain Confusion loss [53]; BL refers to Balancing Loss (i.e., GR or CDC). Models are trained on 5 seeds; error bars show 95% CI.

**Figure 16:**
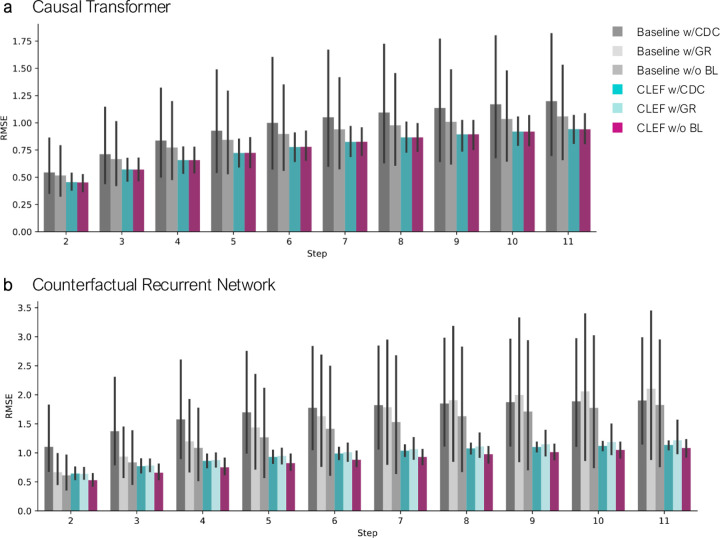
Counterfactual τ-step ahead prediction on semi-synthetic patient trajectories (Appendix C.4). GR refers to Gradient Reversal loss [7]; CDC refers to Counterfactual Domain Confusion loss [53]; BL refers to Balancing Loss (i.e., GR or CDC). Models are trained on 5 seeds; error bars show 95% CI.

**Figure 17:**
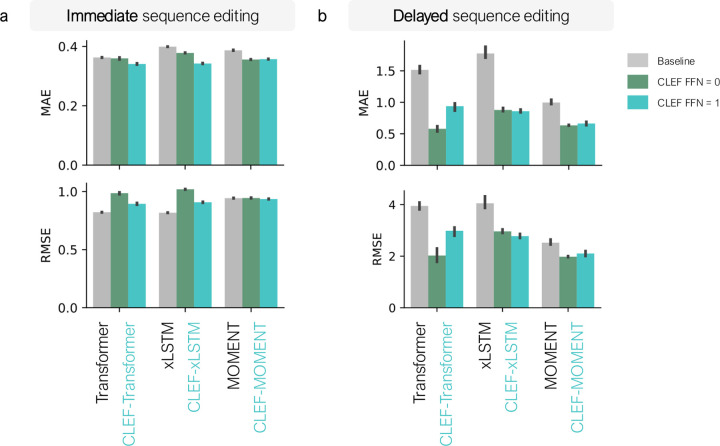
Benchmarking the performance of Clef, baselines, and ablation models on zero-shot **(a)** immediate and **(b)** delayed counterfactual generation of cellular developmental trajectories (Appendix C.1). Performance is measured by MAE (top row) and RMSE (bottom row). Models are trained on 3 seeds; error bars show 95% CI.

**Figure 18:**
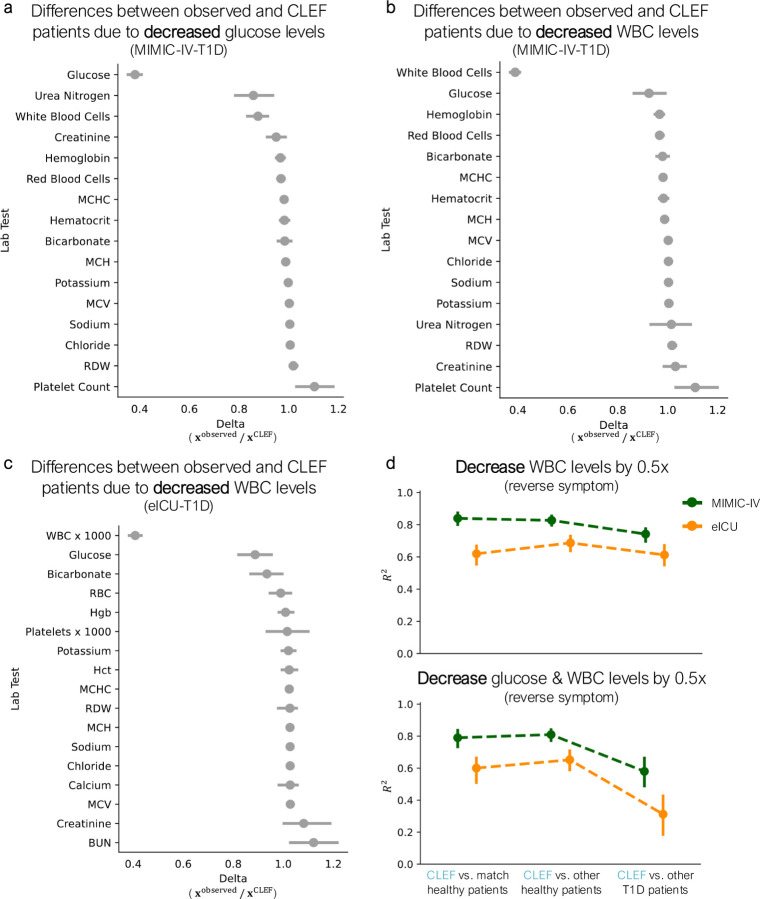
Clef-generated patients via intervention on temporal concepts (Appendix C.2). Observed and Clef patients are compared to quantify the differences between their lab test trajectories as a result of the intervention to halve the **(a)** glucose levels in T1D patients from the MIMIC-IV-T1D cohort, **(b**) white blood cell (WBC) levels in T1D patients from the MIMIC-IV-T1D cohort, and **(c)** WBC levels in T1D patients from the eICU-T1D cohort. **(d)** After intervening on Clef to halve WBC levels, we observe whether the resulting Clef patients’ trajectories are “healthier” or “sicker” compared to other patients in the real-world cohort (top). Further, we investigate whether the intervention effects are compounded when simultaneously reducing glucose and WBC levels by half (bottom). Error bars show 95% CI.

## Footnotes

1 GitHub repository: https://github.com/mims-harvard/CLEF

2 We assume that the condition s always occurs shortly before the measured covariates at time $t_{j}$. When s is an intervention and our problem becomes counterfactual prediction (refer to Section 3.3 for a more rigorous discussion about the formulation of sequence editing in this context), our assumption is consistent with existing practice in the literature of counterfactual prediction.

## A Extended Related Work

### A.1 Leveraging trajectories as inductive biases

Understanding sequential data as trajectories (e.g., increasing, decreasing, constant) is more natural for human interpretation than individual values [38]. Many models on temporal data extract dynamic motifs as inductive biases to improve their interpretability [38, 24, 9]. Such temporal patterns can be used for prompting large pretrained models to perform time series forecasting [9], suggesting that trajectories can capture more universal and transferrable insights about the temporal dynamics in time series data. Trajectories have yet to be adopted for conditional or counterfactual sequence generation.

#### Relevance to Clef.

Temporal concepts c (Definition 3.2) represent trajectories (or rates of change). The concept decoder G leverages temporal concepts c and covariates at the latest time step $x_{:,t_{i}}$ to generate the remainder of the sequence (Section 3.2). To understand how temporal concepts enable Clef models to preserve global consistency: One can think of the latest covariates $x_{:,t_{i}}$ as a set of reference values for each covariate, and these values are modified based on the desired forecast time $t_{j}$ and condition token $z_{s}$. Such modifications are captured by temporal concepts c, which represent the rates of change (or trajectories) for each covariate from time steps $t_{i}$ to $t_{j}$.

### A.2 Building digital twins

Building virtual representations of cells and patients (commonly referred to as virtual cells, virtual patients, or digital twins) has the potential to facilitate preventative and personalized medicine [45, 8]. Medical digital twins (e.g., an artificial lung or pancreas, automated insulin delivery systems, and cardiac twins) have demonstrated clinical utility [45, 40, 76]. There is a wide range of methods for building digital twins, such as mechanistic models (e.g., physics-informed self-supervised learning approach [41]), neural models (e.g., finetuned large language and vision models [52, 3]), and hybrid models (e.g., framework with mechanistic and neural components [28]).

#### Relevance to Clef.

Clef is a machine learning-based neural model. It is a flexible architecture to enable conditional sequence generation (Definition 3.1, Problem Statement 3.1) as well as counterfactual prediction (Section 3.3, Appendix D.2) of continuous multivariate sequences.

### A.3 Additional details

#### Delayed sequence editing vs. long-horizon forecasting.

While long-horizon forecasting and delayed sequence editing both predict the sequence or covariates at a future time $t_{j}$, delayed sequence editing does not require autoregressive predictions from $t_{i}$ to $t_{j}$, which can lead to accumulation of error. Instead, delayed sequence editing allows skipping directly to $t_{j}$ from $t_{i}$ in a single step.

#### Intuition for Clef’s conditional sequence generation architecture.

We leverage the state-of-the-art conditional sequence generation setup [91, 54, 33, 99, 5]. The *sequence encoder* extracts features from historical covariates to learn a hidden representation that captures relevant information for the generation task. Any encoder (e.g., pretrained multivariate foundation model) can be used with Clef. The *condition adapter* projects the condition token to a shared latent space with the sequence and time representations. Because condition tokens are generated by a pretrained foundation model (e.g., ESM2, a protein language model that learns on protein sequences), they capture information that allows Clef to generalize to conditions that have not been observed in the training dataset (e.g., based on shared evolutionary information between protein sequences). The *concept encoder* (Clef only) learns a representation (temporal concepts) that captures information about the condition at the next time step, historical conditions, and historical covariates. The *concept decoder* (Clef only) generates a sequence by applying the learned concepts to the covariates at the last time step $t_{i}$. *With these components*, Clef can generate sequences based on high-dimensional sequences at any future time point and condition. *Without the sequence encoder*, it would be computationally challenging to operate directly on the input historical sequences. *Without the condition adapter*, Clef and state-of-the-art conditional generation models cannot generalize well to unseen conditions in the training dataset. *Without the concept encoder or decoder*, the model may inaccurately generate sequences (refer to Sections 5.1–5.3, and 5.5 for empirical results).

#### Other usage of counterfactuals.

(1) There is extensive work on generating counterfactuals on static data (e.g., single time-step perturbation measured via gene expression profiles or images) [50, 98, 49, 93, 94]. In this work, we focus on longitudinal continuous trajectories. (2) Counterfactual prediction has been used as an additional task to improve the interpretability and accuracy of predictions [95, 26, 90, 48], such as leveraging causal alignment to produce reliable diagnoses [48]. While Clef’s temporal concepts can be intervened upon to interpret model outputs, counterfactual prediction is not an auxiliary task to improve Clef’s interpretability and performance.

#### Excluded baselines for estimating counterfactual outcomes over time.

Causal CPC [18], MambaCDSP [88], and GMCG [2] can estimate counterfactual outcomes over time, but are excluded due to unavailable code. While BNCDE [27] can estimate counterfactual outcomes over time, it is designed to forecast outcomes as well as uncertainty (rather than single-point estimates, which is the focus of Section 3.3). As such, extending BNCDE [27] with Clef is not directly feasible. CF-GODE [32] can estimate continuous-time counterfactual outcomes, but is excluded due to unavailable code.

## B Assumptions for Causal Identification

Under the potential outcomes framework [55, 82] and its extension to time-varying treatments and outcomes [80], the potential counterfactual outcomes over time (i.e., τ-step ahead, where $\tau =t_{j}-t_{i}$, potential outcome conditioned on history from Equation 5) are identifiable from factual observational data under three standard assumptions: consistency, positivity, and sequential ignorability.

**Assumption B.1** (Consistency). Let s be the given sequence of treatments for a patient, consisting of historical treatments $s_{t_{0}:t_{i}}$ and next treatment $s_{t_{j}}$. The potential outcome is consistent with the observed (factual) outcome $x_{:,t_{j}}(s)=x_{:,t_{j}}$.

**Assumption B.2** (Positivity). There is always a non-zero probability of receiving (or not) a treatment for all the history space over time [30]: If $P\left(s_{t_{0}:t_{i}},x_{:,t_{0}:t_{i}}\right)>0$, then $0<P\left(s_{t_{j}}|s_{t_{0}:t_{i}},x_{:,t_{0}:t_{i}}\right)<1$ for all $s_{t_{0}:t_{j}}$. This assumption is also referred to as (sequential) overlap [7, 53].

**Assumption B.3** (Sequential ignorability). The current treatment is independent of the potential outcome, conditioning on the observed history: $s_{t_{j}}⫫x_{:,t_{j}}\left(s_{t_{j}}\right)|s_{t_{0}:t_{i}},x_{:,t_{0}:t_{i}}$. This implies that there are no unobserved confounders that affect both treatment and outcome.

While Assumptions B.2 and B.3 are standard across all methods that estimate treatment effects, they may not always be satisfied in real-world settings [81, 73, 97].

**Corollary B.4** (G-computation). *Assumptions B.1-B. 3 provide sufficient identifiability conditions for* Equation 5 *(i.e., with* G-*computation* [44]). *However, it requires estimating conditional distributions of time-varying covariates* [53]. *Since this could be challenging given a finite dataset size and high dimensionality of covariates, we refrain from the explicit usage of* G-*computation* [53].

Note that the standard setup for counterfactual prediction assumes a fixed time grid and normalized covariates [7, 53]. As such, the standardized data preprocessing pipeline entails forward and backward filling for missing values and standard normalization of continuous time-varying features [7, 53]. With the model architecture shown in Figure 2, these preprocessing steps are not necessary, thereby better reflecting real-world data. Still, Assumptions B.1-B.3 hold for our models depicted in Figure 2.

## C Data & Experimental Setup

We provide further details about data construction, data preparation, and experimental setup. Sections C.1–C.2 and Table 1 describe the novel conditional sequence generation benchmark datasets. Sections C.3–C.4 discuss the standard synthetic and semi-synthetic benchmark datasets for counterfactual outcomes estimation. Each section also contains the corresponding experimental setup. We share code and instructions in our GitHub repository to reproduce the experiments in this paper: https://github.com/mims-harvard/CLEF.

### Overview of novel datasets.

To study cellular development, fibroblast cells derived from mice can be artificially reprogrammed into various other cell states *in vitro*. A cell’s state is defined by its gene expression. Throughout reprogramming, a cell activates transcription factor (TF) genes at different time points to change its gene expression, thereby influencing its developmental trajectory. In Figure 3a, a mouse fibroblast is being reprogrammed over the span of 20 days (D0-D20); color and shape represent cell state. On day 8, if the cell activates the Obox6 TF, the cell is on the path toward becoming an induced pluripotent stem cell (iPSC); whereas if it activates the Neurod4 TF, it is on the path toward becoming a neuron or astrocyte. The health of a human patient is often monitored through lab tests (e.g. blood sodium level, white blood cell count). As shown in Figure 3b, the history of lab results across multiple patient visits (V1-V9) as well as candidate clinical interventions (e.g., medication) can be used to infer the most likely future trajectory of the patient’s health.

**Table 1:** Dataset statistics for conditional sequence generation benchmarks. We construct three core datasets for benchmarking conditional sequence generation: WOT (cellular developmental trajectories), eICU (patient lab tests), and MIMIC-IV (patient lab tests). We also construct a paired counterfactual cellular trajectories dataset, WOT-CF. N is the number of sequences (i.e., cellular developmental trajectories, patient lab test trajectories), V is the number of measured variables (i.e., gene expression, lab test), and L is the length of the sequences.

Dataset	N	V	Mean L	Max L
WOT	3, 000	1, 480	27.03 ± 6.04	37
WOT-CF	2, 546	1, 480	27.01 ± 5.98	37
eICU	108, 346	17	20.27 ± 25.23	858
MIMIC-IV	156, 310	16	15.56 ± 24.43	949

### C.1 Cellular Developmental Trajectories

Here, we describe the process of (1) simulating single-cell transcriptomic profiles of developmental time courses for individual cells and (2) preparing these trajectories for modeling.

#### C.1.1 Simulating trajectories

Cellular reprogramming experiments help elucidate cellular development [83]. In these wet-lab experiments, cells are manipulated and allowed to progress for a specific period of time before they undergo RNA sequencing (RNA-seq), and we analyze the resulting RNA-seq data to observe their new cellular profiles [83]. RNA-seq is a destructive process for the cell, meaning that the same cell cannot be sequenced at two different time points. Computational models are thus necessary to infer the trajectory of a cell.

##### Waddington-OT dataset and model.

Waddington-OT [83] is a popular approach to reconstruct the landscape of cellular reprogramming using optimal transport (OT). There are two components in Waddington-OT: (1) *a single-cell RNA-seq (scRNA-seq*) *dataset* of mouse cells from a reprogramming experiment, and (2) *an OT-based trajectory inference model* fitted on the scRNA-seq dataset. The scRNA-seq dataset consists of 251,203 mouse cells profiled from 37 time points (0.5-day intervals) during an 18-day reprogramming experiment starting from mouse embryonic fibroblasts. The trajectory inference model consists of transport matrices $\pi_{t_{k},t_{k+1}}$ with dimensions N×M that relate all cells $x_{t_{k}}^{1},\ldots ,x_{t_{k}}^{n}$ profiled at time $t_{k}$ to all cells $x_{t_{k+1}}^{1},\ldots ,x_{t_{k+1}}^{m}$ profiled at time $t_{k+1}$. An entry at row i and column j of $\pi_{t_{k},t_{k+1}}$ corresponds to the probability that $x_{t_{k+1}}^{j}$ is a descendant cell of ${\text{x}}_{t_{k}}^{i}$, as determined using optimal transport [11]. Every cell in the scRNA-seq dataset is either pre-labeled as one of the 13 provided cell sets (i.e., induced pluripotent stem, stromal, epithelial, mesenchymal-epithelial transition, trophoblast, spongiotrophoblast, trophoblast progenitor, oligodenrocyte progenitor, neuron, radial glial, spiral artery trophoblast giant, astrocyte, other neural) or unlabeled. We cluster the unlabeled cells using Leiden clustering via scanpy [92] at a resolution of 1, and define the resulting 27 unlabeled clusters as unique cell sets. As a result, each cell in the dataset belongs one and only one cell set.

##### Simulating cell state trajectories.

We define “cell state” as the transcriptomic profile of a cell. Here, a transcriptomic profile is the log-normalized RNA-seq counts of the top 1,479 most highly variable genes. To create a simulated trajectory of cell states for an individual cell undergoing reprogramming, we randomly and uniformly sample a cell profiled at time step $t_{0}$ (Day 0.0) from the Waddington-OT scRNA-seq dataset, and generate via the transport matrix $\pi_{t_{0},t_{1}}$ a probability distribution $P_{t_{1}}$ over possible descendant cells $x_{t_{1}}^{1},\ldots ,x_{t_{1}}^{m}$ at time step $t_{1}$ (Day 0.5). We sample a cell from this distribution, and repeat the process until we reach either Day 18.0 or a terminal state (i.e., neural, stromal, or induced pluripotent stem cell). After generating a trajectory composed of cells from the Waddington-OT scRNA-seq dataset through this process, we retrieve the transcriptomic profile of each cell to compose $x_{:,t_{0}:t_{T}}$, where T is the length of the trajectory.

##### Inferring conditions.

A condition $s_{t_{i}}$ is defined as the activation of a transcription factor (TF) that leads a cell to transition from state $x_{t_{i}}$ to descendant state $x_{t_{i+1}}$. To infer such conditions, we perform differential expression analysis between cells from the same cell set as $x_{t_{i}}$ (i.e., $x^{a}\in A$) and cells from the same cell set as $x_{t_{i+1}}$ (i.e., $x^{b}\in B$). Using the wot.tmap.diff_exp function (via the Waddington-OT library), we identify the top TF that was significantly upregulated in $x^{a}\in A$ compared to $x^{b}\in B$. If no TFs are differentially expressed, then the condition is “None.” We retroactively perform this analysis on all pairs of consecutive cell states in a cell state trajectory $x_{:,t_{0}:t_{T}}$ to obtain the full trajectory containing both cell states and TF conditions: $\tau =\left\{x_{t_{0}},s_{t_{0}},x_{t_{1}},s_{t_{1}},\cdots ,s_{t_{T-1}},x_{t_{T}}\right\}$. In other words, τ represents a simulated trajectory of an individual cell undergoing the reprogramming process. Condition embeddings $z_{s}\in R^{5120}$ are obtained from the (frozen) pretrained ESM-2 embedding model [47].

##### Generating matched counterfactual trajectories.

We additionally create pairs of matched counterfactual trajectories to evaluate a model’s performance in zero-shot counterfactual generation. Each pair consists of an “original” trajectory $\tau_{og}$ and a “counterfactual” trajectory $\tau_{cf}$. First, we generate $\tau_{og}$ using the Waddington-OT model. Then, given a divergence time step D, the first D time steps of $\tau_{og}$ are carried over to $\tau_{cf}$ such that the first D cell states and conditions of $\tau_{og}$ and $\tau_{cf}$ are exactly the same. The remaining states and conditions of $\tau_{cf}$ are sampled independently from $\tau_{og}$, resulting in an alternative future trajectory based on an alternative condition at time step D.

##### Implementation note:

Since Clef learns time embeddings based on the year, month, date, and hour of a given timestamp, we convert the time steps of each cell into timestamps. We set the starting time $t_{0}$ as timestamp 2000/01/01 00:00:00, and add $10\times t_{i}$ hours to the converted timestamp of $t_{i-1}$.

#### C.1.2 Experimental setup

##### Generating data splits.

There are three cell sets (i.e., groups of cells with the same cell state label) that consist of cells from Day 0.0 in our post-clustering version of the Waddington-OT dataset. We refer to these cell sets as “start clusters” because all initial cell states are sampled from one of these cell sets. Since the choice of start cluster can influence the likelihood of a cell’s trajectory reaching certain terminal fates, we split our cellular trajectories into train, validation, and test sets based on their start cluster. This cell-centric data split allows us to evaluate how well a model can generalize to different distributions of trajectories. Start cluster #1 is in the train set, start cluster #3 is in the validation set, and start cluster #2 is in the test set.

##### Zero-shot counterfactual generation.

The data split for zero-shot counterfactual generation is constructed such that the original trajectories $\tau_{og}$ are in the train or validation sets, and the counterfactual trajectories $\tau_{cf}$ are in the test set.

### C.2 Patient Lab Tests

Here, we describe the process of (1) preprocessing electronic health records to extract longitudinal routine lab tests data and (2) preparing these trajectories for modeling.

#### C.2.1 Constructing routine lab test trajectories

We leverage two publicly available medical datasets: eICU [75] and MIMIC-IV [34, 36, 22]. Both datasets are under the PhysioNet Credentialed Health Data License 1.5.0 [74]. The retrieval process includes registering as a credentialed user on PhysioNet, completing the CITI “Data or Specimens Only Research” training, and signing the necessary data use agreements.

We process each dataset (i.e., eICU, MIMIC-IV) separately with the following steps. First, we extract the routine lab tests only (annotation available only in MIMIC-IV) and the most commonly ordered lab tests (i.e., lab tests that appear in at least 80% of patients). Next, we keep patients for whom we have at least one of each lab test. If there are multiple measurements of a lab test at the same time step (i.e., year, month, date, hour, minute, and seconds), we take the mean of its values. We extract patients with more than one visit (or time step).

We define patients’ conditions as medical codes, specifically International Classification of Diseases (ICD), of their diagnosis. Both eICU and MIMIC-IV use ICD-9 and ICD-10 codes. We extract the medical codes and their timestamps (multiple medical codes at a single time step is possible). Since the timestamps of diagnostic codes and lab tests are not necessarily the same (and there are fewer entries of diagnostic codes than lab orders), we merge them with a tolerance range of 12 hours (eICU) or two days (MIMIC-IV). We obtain (frozen) condition embeddings $z_{s}\in R^{128}$ (retrieved on December 22, 2024) from an embedding model that has been pretrained on a clinical knowledge graph [37]. The clinical knowledge graph is constructed by integrating six existing databases of clinical vocabularies used in electronic health records: International Classification of Diseases (ICD), Anatomical Therapeutic Chemical (ATC) Classification, Systemized Nomenclature of Medicine - Clinical Terms (SNOMED CT), Current Procedural Terminology (CPT), Logical Observation Identifiers Names and Codes (LOINC), and phecodes [37].

#### C.2.2 Generating data splits

We generate a standard patient-centric random split for benchmarking model performance (**R1-R2**), and a series of increasingly challenging data splits via SPECTRA [17] to evaluate model generalizability (**R3**). We describe in detail the process of constructing SPECTRA data splits:

SPECTRA [17] creates a series of splits with decreasing cross-split overlap or similarity between the train and test sets. By training and testing models on these splits, we can assess model performance as a function of cross-split overlap (Appendix Figure 10). SPECTRA refers to this relationship as the spectral performance curve, which provides insight into how well a model generalizes to less similar data. When a new dataset split is encountered, it can be plotted as a point on this curve. The area under the spectral performance curve (AUSPC) serves as a metric of model generalizability and enables comparisons across models (Appendix Table 2).

To generate a split with SPECTRA, a similarity definition and a SPECTRA parameter (SP) value between 0 and 1 are required. SP controls the level of cross-split overlap (Appendix Figure 10): values closer to 0 create splits resembling classical random splits, while values closer to 1 produce stricter splits with minimal or no overlap between train and test sets. For example, at an input of 1, no similar samples are shared between the train and test sets.

For eICU and MIMIC-IV, we define two patients as similar if: (1) they are of the same gender, (2) they are born in the same decade, and (3) they share at least one ICD-9 or ICD-10 category. We exclude ICD-9 and ICD-10 codes that are present in more than 50% of patients to avoid overly generic features. SPECTRA systematically prunes similar patients to produce splits. For this study, we generate 20 splits with SP values that are evenly spaced between 0 and 1 (Appendix Figure 10). Given a train and test set, cross-split overlap is defined as the proportion of samples in the train set that are similar to at least one sample in the test set (Appendix Figure 10).

#### C.2.3 Constructing cohorts of patients with type 1 diabetes mellitus

We conduct case studies on two independent cohorts of patients with type 1 diabetes mellitus (T1D), a chronic autoimmune disease in which the immune system attacks insulin-producing cells in the pancreas [77]. From our processed eICU and MIMIC-IV datasets, we construct two cohorts of T1D patients and matched healthy individuals.

##### Procedure.

To define a type 1 diabetes mellitus (T1D) patient cohort in eICU and MIMIC-IV, we identify patients with T1D and matched healthy individuals. A patient has T1D if the ICD-10 code E10 (or the equivalent ICD-9 code 250) is present in the electronic health records. Matched healthy patients are defined by three criteria. First, the patient must not contain any of the following ICD-10 (and ICD-9 equivalent) codes: E11, E13, E12, E08, E09, R73, and 024. An initial healthy patient cohort is constructed using these filtering codes. Next, we identify frequently co-occurring ICD codes between the initial set of patients and patients with T1D to filter out generic ICD codes (threshold = 20). Finally, healthy patients are matched with a T1D patient if: they are of the same gender, they are born in the same decade, and they share at least 50% of ICD codes.

##### Data statistics.

eICU-T1D contains 59 T1D patients and 579 matched healthy controls, while MIMIC-IV-T1D includes 25 T1D patients and 226 matched healthy controls.

#### C.2.4 Experimental setup for type 1 diabetes mellitus case study

We evaluate Clef’s ability to simulate counterfactual patient trajectories through temporal concept intervention. This is analogous to intervening on concept bottleneck models by editing concept values and propagating the changes to the final prediction [39]. Such a capability is particularly valuable when condition tokens are insufficient, such as when prescribing medication dosage. Editing concept values allow users, such as clinicians, to simulate potential trajectories as a result of the precise edits.

We conduct case studies on two independent cohorts of patients with type 1 diabetes mellitus (T1D) (Appendix C.2.4). To generate counterfactual sequences, we modify specific values in temporal concept c, such as glucose levels, and allow Clef to simulate future trajectories of length T=10. We then compare these counterfactual trajectories (i.e., Clef-generated patients) against observed sequences from matched healthy individuals, other healthy individuals, and other T1D patients. Our hypothesis is that clinically meaningful edits will produce “healthier” (i.e., more similar to healthy patients) or “sicker” (i.e., more similar to other T1D patients) trajectories.

### C.3 Synthetic tumor growth trajectories

The tumor growth simulation model [21] produces trajectories of tumor volume (i.e., one-dimensional outcome) after cancer diagnosis. There are two binary treatments (i.e., radiotherapy $A_{t}^{r}$ and chemotherapy $A_{t}^{c}$) at time t, and the possible treatments are: $\left\{\left(A_{t}^{c}=0,A_{t}^{r}=0\right),\left(A_{t}^{c}=1,A_{t}^{r}=0\right)\right.,\left.\left(A_{t}^{c}=0,A_{t}^{r}=1\right),\left(A_{t}^{c}=1,A_{t}^{r}=1\right)\right\}$. For τ-step ahead prediction, we simulate synthetic tumor growth trajectories under single-sliding treatment (i.e., shift the treatment over a window) [7, 53] and random trajectories (i.e., randomly assign treatments) settings [53]. Importantly, the ground-truth counterfactual trajectories are known. We limit the length of trajectories to a maximum of 60 time steps. For each setting, we generate trajectories with different amounts of time-varying confounding γ∈[0,1,2,3,4], each with 10,000 trajectories for training, 1,000 for validation, and 1,000 for testing.

We follow the data simulation process and experimental setup as described in Appendix J and GitHub repository of the original Causal Transformer publication [53].

### C.4 Semi-synthetic patient trajectories

MIMIC-III-CF is a semi-synthetic dataset based on patient data from real-world intensive care units [89, 35]. The data are aggregated at hourly levels, with forward and backward filling for missing values and standard normalization of the continuous time-varying features [89, 35, 7, 53]. Patients have 25 different vital signs as time-varying covariates and 3 static covariates (gender, ethnicity, age). Untreated trajectories of outcomes are first simulated under endogenous and exogenous dependencies, and then treatments are sequentially applied [53]. There are 3 synthetic binary treatments and 2 synthetic outcomes [53]. Importantly, the ground-truth counterfactual trajectories are known. We limit the length of trajectories to a maximum of 60 time steps. We generate 1,000 patients into train, validation, and test subsets via a 60%, 20%, and 20% split. For τ-step ahead prediction with $\tau_{\text{max}}=10$, we sample 10 random trajectories for each patient per time step.

We follow the data simulation process and experimental setup as described in Appendix K and GitHub repository of the original Causal Transformer publication [53].

## D Implementation Details

We provide code and instructions in our GitHub repository to implement Clef, baselines, and ablations: https://github.com/mims-harvard/CLEF. To implement baselines, we follow the authors’ recommendations on model design and hyperparameter selection from the original publications. We do not share data or model weights that may contain sensitive patient information.

### D.1 Baselines for Conditional Sequence Generation

We benchmark Clef against the state-of-the-art conditional sequence generation setup with different sequential data encoders (Figure 2): Transformer [91, 54, 33, 99] and xLSTM [5]. We further compare against a state-of-the-art time series foundation model, MOMENT [24], as the sequence encoder. Specifically, we finetune an adapter for the 1024-dimensional embeddings from the frozen MOMENT-1-large embedding model.

### D.2 Clef Extensions and Baselines for Counterfactual Outcomes Estimation

Due to its versatility, Clef can be leveraged by state-of-the-art machine learning models designed to estimate counterfactual outcomes [7, 53]. Counterfactual Recurrent Network (CRN) [7] and Causal Transformer (CT) [53] demonstrate state-of-the-art performance in the established benchmarks [7, 53]. To implement Clef-CRN and Clef-CT, the GELU activation layer from the concept encoder E (Equation 2) and the concept decoder G (Equation 3) of Clef are appended to outcome predictor network (denoted as $G_{Y}$ where Y is the outcome of the given treatment in the original publications [7, 53]) of CRN and CT. Following the original CRN and CT publications, we minimize the factual outcome loss (i.e., output of $G_{Y}$) via mean squared error (MSE) [7, 53].

We evaluate Clef against their non-Clef counterparts with and without balancing loss functions (i.e., gradient reversal (GR) [7], counterfactual domain confusion (CDC) loss [53]). This results in 5 distinct state-of-the-art baselines: CRN with GR loss (i.e., original CRN implementation) [7]; CRN with CDC loss [53]; CRN without balancing loss [53]; CT with CDC loss (i.e., original CT implementation) [53]; and CT without balancing loss [53].

### D.3 Model Training

Models are trained on a single NVIDIA A100 or H100 GPU. All models have comparable number of parameters as their Clef-based counterparts.

### D.4 Hyperparameter Sweep

The selection of hyperparameters for the (conditional sequence generation) models trained from scratch are: dropout rate ϵ [0.3, 0.4, 0.5, 0.6], learning rate ϵ [0.001, 0.0001, 0.00001], and number of layers (or blocks in xLSTM) ϵ [4,8]. Because the number of heads must be divisible by the number of features, the number of heads for eICU (18 lab tests) ϵ [2, 3, 6, 9] and for others ϵ [4, 8]. For xLSTM, the additional hyperparameters are: 1D-convolution kernel size ϵ [4, 5, 6] and QVK projection layer block size ϵ [4, 8].

### D.5 Best Hyperparameters

#### MIMIC-IV dataset.

The best hyperparameters for the (conditional sequence generation) models trained on the MIMIC-IV dataset are: dropout rate = 0.6, learning rate = 0.0001, number of layers (blocks in xLSTM) = 8, and number of heads = 4. For xLSTM models, 1D-convolution kernel size = 4 and QVK projection layer block size = 4. For Clef models, the number of FNN in the concept encoder = 1 (Appendix Figures 8–9).

#### eICU dataset.

The best hyperparameters for the (conditional sequence generation) models trained on the eICU dataset are: dropout rate = 0.6, learning rate = 0.0001, number of layers (blocks in xLSTM) = 8, and number of heads = 6. For xLSTM models, the number of heads = 2, 1D-convolution kernel size = 4 and QVK projection layer block size = 4. For Clef models, the number of FNN in the concept encoder = 1(Appendix Figures 8–9).

#### Waddington-OT (WOT) dataset.

The best hyperparameters for the (conditional sequence generation) models trained on the WOT dataset are: dropout rate = 0.6, learning rate = 0.00001, number of layers (or blocks in xLSTM) = 4, number of heads = 8. For xLSTM models, 1D-convolution kernel size = 4 and QVK projection layer block size = 8. For CleF models, the number of FNN in the concept encoder = 0 (Appendix Figures 8–9).

#### Synthetic tumor growth and semi-synthetic patient trajectories datasets.

For the counterfactual prediction models (including Clef and non-Clef models), we follow the best hyperparameters as reported in the original publications of CT [53] and CRN [7].

## E Additional Figures and Tables

**Table 2:** Generalizability of Clef, baselines, and ablations on eICU and MIMIC-IV datasets (Appendix C.2) in immediate and delayed sequencing. Performance is measured by the area under the spectral performance curve (AUSPC) for MAE (Appendix Figure 11) or RMSE (Appendix Figure 12). Smaller AUSPC values indicate better performance. Models are trained on 3 seeds; standard deviation is reported.

Model	eICU				MIMIC-IV			
	Immediate		Delay		Immediate		Delay	
	MAE	RMSE	MAE	RMSE	MAE	RMSE	MAE	RMSE
Transformer	27.06 ± 0.98	59.83 ± 1.14	22.59 ± 1.21	50.29 ± 0.56	40.87 ± 0.15	71.77 ± 0.21	44.61 ± 0.19	80.38 ± 0.32
+ Clef	15.16 ± 1.09	32.95 ± 2.47	14.36 ± 1.07	34.27 ± 2.12	32.79 ± 1.41	57.76 ± 3.39	35.65 ± 1.73	65.10 ± 4.43
+ Clef + FFN	10.99 ± 0.31	27.57 ± 0.27	9.25 ± 0.60	27.69 ± 0.22	21.35 ± 3.16	36.92 ± 5.46	23.83 ± 3.26	44.11 ± 5.83
xLSTM	28.47 ± 0.63	62.28 ± 1.38	23.11 ± 0.91	52.53 ± 1.98	40.75 ± 0.30	71.90 ± 0.40	44.31 ± 0.24	80.38 ± 0.33
+ Clef	16.73 ± 2.16	35.43 ± 6.01	15.32 ± 2.10	34.68 ± 7.09	32.06 ± 1.13	53.42 ± 2.18	33.88 ± 1.98	57.73 ± 3.63
+ Clef + FFN	11.35 ± 0.11	28.09 ± 0.08	9.04 ± 0.18	26.21 ± 0.48	21.04 ± 2.32	37.50 ± 4.60	22.63 ± 2.61	42.12 ± 5.03
MOMENT	53.49 ± 0.03	90.54 ± 0.03	48.83 ± 0.02	82.50 ± 0.02	46.55 ± 0.01	77.22 ± 0.01	50.59 ± 0.02	85.72 ± 0.01
+ Clef	47.69 ± 0.33	82.18 ± 0.34	40.10 ± 0.44	72.70 ± 0.46	44.01 ± 0.35	73.83 ± 0.63	46.88 ± 0.38	81.20 ± 1.27
+ Clef + FFN	47.56 ± 1.60	82.81 ± 2.88	39.91 ± 1.65	72.54 ± 3.20	42.92 ± 0.52	70.72 ± 1.96	45.75 ± 0.65	77.35 ± 2.77

## F Limitations & Future Directions

There are two key limitations of Clef. Firstly, we define temporal concepts such that each element represents a unique measured variable in the sequence (e.g., gene expression, lab test). Instead, it may be beneficial to learn higher-order relationships between the measured variables or across time as abstract hierarchical concepts [86, 38]. Secondly, while Clef is able to generate counterfactual sequences for any condition, including those it may not have seen during training, Clef could potentially improve with additional guidance from a real-world causal model for the system or domain of interest [10]. Since defining such a real-world causal graph is a major challenge, one promising future direction could be to enable user interventions, such as those performed in our T1D case studies, to finetune Clef. While this work focuses on cellular and patient trajectories, Clef can be readily extended to perform sequence editing in other domains.

## G Broader Impact

By introducing a flexible and interpretable approach to conditional sequence generation, Clef bridges the gap between language model-style conditional generation and structured, time-sensitive sequence editing, with implications for decision support in medical and scientific applications. Like all generative AI models, Clef (and its derivatives) should be used solely for the benefit of society. In this study, we show that Clef can generate alternative cellular trajectories and simulate the reversal or progression of symptoms to model healthier or sicker patient outcomes. However, this work (and any derivatives) should never be used to induce harmful cellular states (e.g., activating transcription factors to drive a cell toward a pathological state) or negatively impact patient care (e.g., neglecting necessary clinical interventions or recommending harmful treatments). Our goal is to help researchers understand the underlying mechanisms of disease to improve public health. Any misuse of this work poses risks to patient well-being. Therefore, the ability to intervene on Clef’s generated outputs should be leveraged to assess the model’s robustness and correctness for ethical and responsible use.
